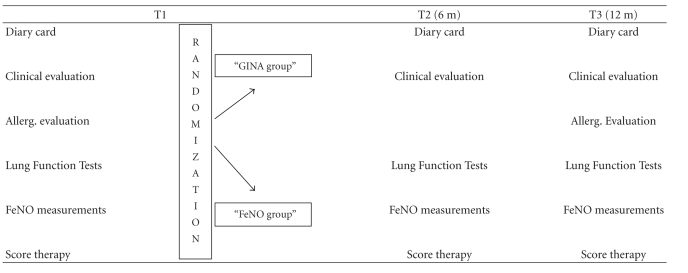# FeNO as a Marker of Airways Inflammation: The Possible Implications in Childhood Asthma Management

**DOI:** 10.1155/2010/691425

**Published:** 2010-05-18

**Authors:** Marcello Verini, Nicola Pietro Consilvio, Sabrina Di Pillo, Anna Cingolani, Cynzia Spagnuolo, Daniele Rapino, Alessandra Scaparrotta, Francesco Chiarelli

**Affiliations:** ^1^Allergological and Pneumological Service, Department of Pediatric, University “G. D'Annunzio”, 66100 Chieti, Italy; ^2^Department of Pediatric, University “G. D'Annunzio”, 66100 Chieti, Italy

## Abstract

The aim of this study was to verify FeNO usefulness, as a marker of bronchial inflammation, in the assessment of therapeutic management of childhood asthma. We performed a prospective 1-year randomized clinical trial evaluating two groups of 32 children with allergic asthma: “GINA group”, in which therapy was assessed only by GINA guidelines and “FeNO group”, who followed a therapeutic program assessed also on FeNO measurements. Asthma Severity score (ASs), Asthma Exacerbation Frequency (AEf), and Asthma Therapy score (ATs) were evaluated at the start of the study (T1), 6 months (T2), and 1 year after (T3). *ASs* and *AEf* significantly decreased only in the FeNO group at times T2 and T3 (*p[T1-T2] = *0.0001, and *p[T1-T3] = *0.01; *p[T1-T2] = *0.0001; and *p[T1-T3] < *0.0001, resp.). After six months of follow-up, we found a significant increase of patients under inhaled corticosteroid and/or antileukotrienes in the GINA group compared to the FeNO group (*P* = .02). Our data show that FeNO measurements, might be a very useful additional parameter for management of asthma, which is able to avoid unnecessary inhaled corticosteroid and antileukotrienes therapies, however, mantaining a treatment sufficient to obtain a meaningful improvement of asthma.

## 1. Introduction

Asthma is characterized by variable degrees of airway obstruction, hyperresponsiveness, and chronic inflammation [1]. Current guidelines emphasize that inhaled corticosteroids (ICSs) represent the main treatment for asthma because they target the underlying airways inflammation [2].

Actually decisions to start ICSs and/or long-acting *β*2-adrenergic agonist (LABA) and/or antileukotrienes (a-LT), or change the dose are mainly based on symptoms reported by the child or parents [3]. Nevertheless symptoms are nonspecific and not closely related to the presence and severity of airways inflammation [4]. Lung function tests show only marginal correlation with airways inflammation [5].

Bronchial epithelium produces Nitric Oxide (NO) [6], and its fraction in exhaled air (FeNO) is elevated in atopic asthma and reflects eosinophilic airways inflammation [7]. Many studies have shown that allergological markers correlate with FeNO levels, and particularly elevated FeNO levels have been found mainly in atopic than in nonatopic asthma [8–10].

Therefore, measurement of FeNO represents a noninvasive marker that may be a useful guide for the adjustment of ICSs treatment [11]. The hypothesis of this study was to verify if FeNO measure is useful in terms of better asthma management in children. The aim of our study was to examine whether the inclusion of repeated FeNO measurements into asthma monitoring leads to an improvement in asthma outcome, with an exacerbation reduction, clinical improvement, and therapy score reduction.

## 2. Subjects

This was a prospective randomized study. We recruited 64 Caucasian children (36 males and 28 females; aged between 6 and 17 years) who had been referred to the Allergological and Pneumological Unity of the Pediatric Department, University of Chieti, Italy, between January 2005 and January 2006. All subjects had been admitted for allergic asthma and the diagnosis was made by a pediatric respiratory physician on the basis of clinical history of repeated episodes of coughing, dyspnea, and wheezing, according to ATS-ERS criteria [12].

Patients were randomly allocated to two groups (Table 1):

- “FeNO group” → 32 children (18 boys and 14 girls; mean age, 10.7 ± 2.4 years)
- “GINA group” → 32 children (18 boys and 14 girls; mean age, 11.3 ± 2.1 years).

The whole study population was assessed at baseline (T1), after a period of six months (T2), and at the end of 1-year followup (T3) (Table 2).

At baseline and at 6 months, in the GINA group, therapy was based on symptoms, short acting *β*2-agonist use, and lung function, according to GINA guidelines [13], while in the FeNO group, therapy was assessed according also to FeNO measurements.

The study was approved by the Ethical Committee of the University of Chieti. Written informed consent was obtained from all parents and oral consent from all children.

## 3. Methods

Asthma Severity score (ASS), Asthma Exacerbation frequency (AEF), Asthma Therapy score (ATS), and immunoallergological and functional data were evaluated at the start of the study (T1), 6 months (T2), and 1 year later (T3).

Asthma was classified according to GINA guidelines to: Intermittent Asthma, Mild Persistent, Moderate Persistent, and Severe Persistent Asthma, considering the 6 months before the beginning of the study [13]. 

### 3.1. Asthma Severity Score (ASS)

It was assessed as

- Intermittent Asthma = Score 1,
- Mild and Moderate Persistent Asthma = Score 2,
- Severe Persistent Asthma = Score 3.

We also assessed an arbitrary implementation of asthma classification by GINA criteria, and performed the following score for these two phenotypes of asthma:

- Remission Asthma = Score 0,
- Exercise induced Asthma = Score 2.

### 3.2. Asthma Exacerbation Frequency (AEF)

It was assessed with an anamnestic questionnaire evaluating frequency of asthma exacerbation (defined as the number of episodes of coughing, dyspnea, and wheezing, according to ATS-ERS criteria [12], requiring short-acting *β*2-adrenergic agonist) during the 6 months before the beginning of the study, between T1 and T2 and between T2 and T3 evaluations.

### 3.3. Asthma Therapy Score (ATS)

It was assessed with an anamnestic questionnaire and an arbitrary score, as follows:

- Antihistamines, Ketotifen, Cromones = Score 1,
- Specific Immuno Therapy (SIT), long-acting *β*2-adrenergic agonist (LABA) or antileukotrienes (a-LT) = Score 2,
- ICSs = Score 3.

### 3.4. Allergic Sensitization

It was evaluated by Skin Prick Test (SPT) and serum-specific IgE measurements for the most common respiratory allergens: Dust Mite (Dermatophagoides Pteronyssinus, and Farinae), Grass, Parietaria, Artemisia Vulgaris, Olive, Cypress, Lime, Stone, Elm, Plane, Cat and Dog dander, Alternaria Alternata, and Aspergillus Fumigatus (moulds). Determination of Allergen-Specific IgE was made by an Immunoenzymatic Allergo-sorbent Test (Cap test Pharmacia) [14, 15].

### 3.5. Inflammatory Cells

Peripheral blood eosinophil counts and serum eosinophil cationic protein (s-ECP) levels were measured by immunofluorescence.

### 3.6. Respiratory Function

Evaluations were made by Flow/Volume, curves, Static Lung Volume and Plethysmographic Airway Resistances determination according to ATS/ERS Guidelines [16, 17].

### 3.7. Respiratory Inflammation

All children of the FeNO group underwent FeNO analysis. FeNO was determined with an on-line method using a single breath exhalation and a sensitive chemiluminescence assay (Ecomedics CLD 88), according to ATS-ERS [18]. Patients made an inspiration of eNO-free air via a mouthpiece immediately followed by full exhalation at a constant rate (50 mL/sec) for at least 5 seconds. The mean of three readings at the end of the expiration (plateau phase) was taken as the representative value for each measurements. 12 ppb or more were considered elevated values, according to ATS-ERS criteria [19].

## 4. Statistical Analysis

All values were expressed as means and SD. We analysed differences in variables (ASS, ATS, AEF, and respiratory function test) obtained at different times between the two groups by unpaired T-test, and within each group with paired T-test. Statistical significance level was *P* < .05.

The comparison of the differences in AEF and in ATS between the two groups was calculated also using the Chi square (*χ* ^2^) Test.

The number of antiasthmatic drugs used in the two groups was calculated at T1, T2, and T3.

In the “FeNO group”, level of 12 ppb was the cut-off used to guide the therapeutic management [at 6 months of the follow-up study (T2)], according to the ERS-ATS Statement of 2001 [19].

Values above 12 ppb were considered as an indication to increase the number of drugs, whereas values below 12 ppb lead to a reduction or to a maintenance in the amount of drugs.

## 5. Results

**Figure 1:**
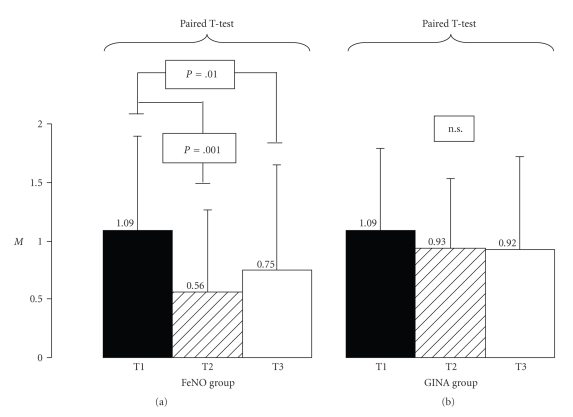
Asthma Severity score (ASS) in FeNO and GINA groups at the first evaluation (T1) after 6 months (T2) and after 1 year of therapy (T3). Mean Values ± SD.

The two groups were similar for age, sex, weight, and height without significant differences. All the children of both groups were allergic to Dermatophagoides Pteronyssinus (Df. pt). At the first assessment, patients' asthma characteristics were similar between the two groups. Asthma Severity score (ASS), Asthma Exacerbations frequency (AEF), Asthma Therapy score (ATS), functional and immunoallergological (circulating eosinophils, s-ECP, total and specific IgE) parameters did not show any significant difference between the two groups at T1. ASS mean values significantly decrease in the FeNO group at T2 and T3 (from 1.09 ± 0.81 to 0.56 ± 0.75 at T2, *p*[*T1-T2*]* = 0.001*, and to 0.75 ± 0.95 at T3, *p*[*T1-T3*]* = 0.01*, resp.), while no difference was detected in the GINA group in the corresponding times (from 1.09 ± 0.77 to 0.93 ± 0.61 at T2, p[T1-T2] = 0.1, and to 0.92 ± 0.82 at T3, p[T1-T3] = 0.1) (Figure 1).

**Figure 2:**
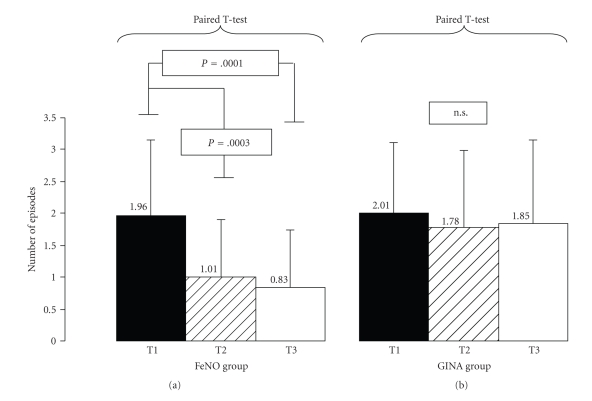
Asthma Exacerbation frequency (AEF) in the FeNO and GINA groups at the first evaluation (T1) after 6 months (T2) and after 1 year of therapy (T3). Mean Number of episodes ± SD.

**Figure 3:**
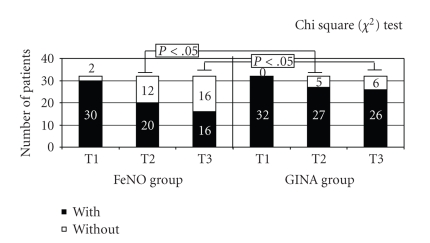
Number of patients with and without asthma exacerbation in FeNO and GINA groups at first evaluation (T1), after 6 months (T2) and after 1 year of therapy (T3).

Similarly, AEF evaluation showed a significant reduction in the number of episodes in the FeNO group at T2 and T3 (Mean values: from 1.96 ± 1.18 to 1.01 ± 0.96 at T2, *p*[*T1-T2*]* = 0.0003*, and to 0.83 ± 0.98 at T3, *p*[*T1-T3*]* = 0.0001*) but not in the GINA group (Mean values: from 2.01 ± 1.17 to 1.78 ± 1.29 at T2, p[T1-T2] = 0.08; and to 1.85 ± 1.34 at T3, p[T1-T3] = 0.14) (Figure 2). Using the Chi-square (*χ* ^2^) Test we found a significant decrease of the number of patients with asthma exacerbation only in the FeNO group at T2 (*P* = .0006) and at T3 (*P* < .05) (Figure 3).

**Figure 4:**
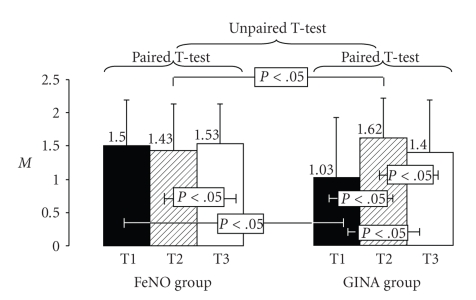
Antiinflammatory drugs level in both group at each evaluation. Mean Values ± SD.

 In addition in the FeNO group, there was no increase in antiasthmatic therapy (Mean values of ATS were similar in the 3 evaluations), (1.5 ± 0.7 in T1; 1.43 ± 0.7 in T2, and 1.53 ± 0.6 in T3) while in the GINA group, the treatment was significantly step up (Mean values: from 1.03 ± 0.9 to 1.62 ± 0.6 at T2, *p*[*T1-T2*]* < 0.05*, and to 1.4 ± 0.7 at T3, *p*[*T2-T3*]* < 0.05; p*[*T1-T3*]* < 0.05*), as shown in Figure 4.

**Figure 5:**
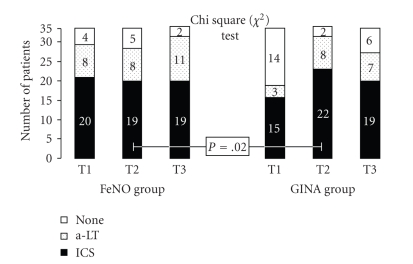
Antiinflammatory drugs trend: variation of the number of patients that use antiinflammatory drugs.

Using the Chi square (*χ* ^2^) Test, we found a significative difference in the number of patients who did not use ICS and/or a-LT after 6 months between the FeNO group (5/32 = 15%) in comparison to the GINA group (2/32 = 6%) [T2: *P* = .02]. No significant difference was found between the two groups at the end of the study; nevertheless, a careful observation relative to the therapy revealed a light difference among the two groups about the anti-inflammatory drugs use. In fact, the FeNO group keeps the same therapy, while the GINA group showed an increased use of ICSs and a-LT (Figure 5).

The evaluation of FeNO levels in FeNO group demonstrated a significant reduction after 6 months of therapy, with a return at the levels of the beginning at the end of the study (Mean values of FeNO: from 13.78 ± 12.31 to 9.51 ± 11.04 at T2, *p*[*T1-T2*]* = 0.0006*, and to 13.53 ± 10.74 at T3, *p*[*T2-T3*]* = 0.005*; p[T1-T3] = 0.44).

No significant difference was found between the two groups in terms of respiratory function test and of imunoallergological parameters.

## 6. Discussion

This study shows that FeNO measurements are useful in childhood asthma management because FeNO reductions are related to improvement in clinical score with a reduction of asthma exacerbation. In our study, FeNO reductions were not related to any improvement of respiratory function but allow to keep the same therapeutic regimen in the “FeNO group”, while in the “GINA group”, we had a significant increase of number of patients who use antiinflammatory drugs (mainly ICS) without any evidence of increase in bronchial inflammation.

Previous studies have shown a significant correlation between FeNO and respiratory symptoms, bronchial hyperresponsiveness (BHR), and blood eosinophilia but not with spirometric indices of lung function [20, 21]. These findings are in agreement with data reported by Silvestri and coworkers [22] who demonstrated that airways inflammation may not be strictly related to a reduction in lung volumes or to the degree of airflow limitation.

Recent studies revealed that FeNO is a potentially useful measure to evaluate the role of airways inflammation in asthma, as it represents the forerunner of an important event in asthma: the remodelling of bronchial airway [23]. 

Current guidelines recommended to adjust the dose of these drugs on the basis of symptoms and LFTs results [13]. However, recent studies demonstrated that the use of alternative criteria, like BHR, FeNO levels, and sputum eosinophils, leads to an improvement in asthma treatment outcomes [24].

The ICSs therapy produces a rapid reduction (dose-dependent) of FeNO levels in asthmatic subjects. Some studies demonstrated that the reduction of FeNO levels occurs at the same time of the reduction of sputum eosinophils [25].

The elevated sensitivity of FeNO levels to ICSs therapies shows that it can be used as a factor of prediction for anti-inflammatory therapy, and could be an indicative marker to detect patients that do not have a good compliance to therapy. Also the anti-LT drugs association could improve FeNO levels, while LABA does not reduce it [26, 27]. Therefore, exhaled nitric oxide may be a valuable parameter to monitor adherence to steroids, although it is less suitable to describe physiologically relevant impairments of lung function [28].

In our study, we found a reduction of clinical symptoms and asthma exacerbations in the FeNO group. Furthermore, we found also an improvement of respiratory function and therapeutic score. We demonstrated that measurement of airways inflammation is of practical value in management of asthmatic children because it allows to monitor patients from a “flogistic” point of view. It leads to an improvement of clinical outcome and to a reduction in number of exacerbations. Furthermore, differently from other studies, in our study FeNO measurement did not permit to reduce the use of drugs but allows us to not increase the amount of drugs as it has been done in GINA group. In particular, we underlied a mild increase of therapeutic score in FeNO group due to an increase of drugs dose to reach a good clinical outcome. Only a few longitudinal studies have examined the possible clinical relevance of FeNO in asthma management. Roberts and coworkers [29] have demonstrated that FeNO relates to previous allergen exposure and asthma control. Pijnenburg et al. [30] demonstrated that FeNO is helpful in predicting asthma relapse in children who discontinue ICSs because of clinical remission. Similarly, the study of Zacharasiewicz et al. [2] performed on 40 children with stable asthma eligible for inhaled steroid reduction has showed that elevated values of sputum eosinophil and FeNO were a significant predictor for failed ICSs reduction in children with apparently well-controlled asthma. These findings suggest that monitoring airway inflammation may be useful in optimizing treatment in childhood asthma. In a recent single-blind controlled trial in adult with asthma, Smith and colleagues [31] showed that using FeNO for adjustments of ICSs leads to similar asthma control with less ICS in the FeNO-treated group compared with the group treated on conventional parameters; the FeNO levels can offer a method to adjust the doses of ICSs. Indeed, use of FeNO measurements may also help to minimize the potential long-term side effects related to ICSs, which are more likely when higher doses are used. Also in the clinical trial of Pijnenburg et al. [3], performed on 85 children with asthma, 1 year of steroids titration FeNO did not result in higher steroid doses and did improve airway hyperresponsiveness and inflammation compared with titrating on symptoms only.

## 7. Conclusion

Our results have shown that the use of noninvasive methods to monitor airway inflammation, as FeNO measure, can help to guide treatment in childhood asthma management. We demonstrate that repeated FeNO measurement can help to optimize asthma therapy with improvement in asthma severity and exacerbation. These findings confirm the important role of FeNO in the routine assessment of children with asthma in clinical practice, especially when decisions about treatment need to be made. Overall, this approach offers a logical complementary item to the use of clinical and functional data leading to a more fitted treatment in childhood asthma management to reach a good clinical outcome and a reduction of exacerbations. Our study shows that FeNO measurement does not allow only a reduction of drugs use but also a better personal fitted therapy sufficient to obtain good clinical control.

Further prospective studies on lager populations of children are required to confirm these conclusions.

## Figures and Tables

**Table 1:** Clinical characteristics of the population.

Characteristics	“FeNO group”	“GINA group”
M/F	18/14	18/14
Age (years)	10.7 ± 2.4	11.3 ± 2.1
Weight (Kg)	45.7	47.5
Height (cm)	149	152
Remission Asthma	7	7
Intermittent Asthma	18	19
Persistent Asthma	7	6
Asthma Duration (years)	5.7 ± 2.61	5.75 ± 2.27

**Table 2:** Study design.